# A crosstalk between gut and brain in sepsis-induced cognitive decline

**DOI:** 10.1186/s12974-022-02472-4

**Published:** 2022-05-23

**Authors:** Vijayasree V. Giridharan, Jaqueline S. Generoso, Leonardo Lence, Gabriela Candiotto, Emílio Streck, Fabricia Petronilho, Anilkumar Pillai, Tarek Sharshar, Felipe Dal-Pizzol, Tatiana Barichello

**Affiliations:** 1 Faillace Department of Psychiatry and Behavioral Sciences, McGovern Medical School, The University of Texas Health Science Center at Houston (UTHealth), Houston, TX USA; 2 Laboratory of Experimental Neurology, Graduate Program in Health Sciences, University of Southern Santa Catarina (UNESC), Criciúma, SC Brazil; 3 Laboratory of Neurometabolic Diseases, Graduate Program in Health Sciences, University of Southern Santa Catarina (UNESC), Criciúma, SC Brazil; 4 Pathophysiology of Neuropsychiatric Disorders Program, Faillace Department of Psychiatry and Behavioral Sciences, McGovern Medical School, The University of Texas Health Science Center at Houston (UTHealth), Houston, TX USA; 5 Neuroscience Graduate Program, The University of Texas MD Anderson Cancer Center UTHealth Graduate School of Biomedical Sciences, Houston, TX USA; 6 Research and Development, Charlie Norwood VA Medical Center, Augusta, GA USA; 7 Department of Psychiatry and Health Behavior, Augusta University, Augusta, GA USA; 8 GHU Paris Psychiatrie et Neuroscience, Neurointensive Care and Neuroanesthesia Department, Paris, France; 9 Université de Paris Cité, Paris, France; 10 Institute of Psychiatry and Neurosciences of Paris, NSERM UMR 1266, Paris, France; 11 Laboratory of Experimental Pathophysiology, Graduate Program in Health Sciences, University of Southern Santa Catarina (UNESC), Criciuma, SC Brazil

**Keywords:** Sepsis, Inflammation, Microglia, Astrocyte, Cytokines, Microbiome, PET, TSPO, Behavior

## Abstract

**Background:**

Sepsis is a potentially fatal disease characterized by acute organ failure that affects more than 30 million people worldwide. Inflammation is strongly associated with sepsis, and patients can experience impairments in memory, concentration, verbal fluency, and executive functioning after being discharged from the hospital. We hypothesize that sepsis disrupts the microbiota–gut–brain axis homeostasis triggering cognitive impairment. This immune activation persists during treatment, causing neurological dysfunction in sepsis survivors.

**Methods:**

To test our hypothesis, adult Wistar rats were subjected to cecal–ligation and perforation (CLP) or sham (non-CLP) surgeries. The animals were subjected to the [^11^C]PBR28 positron emission tomography (PET)/computed tomography (CT) imaging at 24 h and 10 days after CLP and non-CLP surgeries. At 24 h and 10 days after surgery, we evaluated the gut microbiome, bacterial metabolites, cytokines, microglia, and astrocyte markers. Ten days after sepsis induction, the animals were subjected to the novel object recognition (NOR) and the Morris water maze (MWM) test to assess their learning and memory.

**Results:**

Compared to the control group, the 24-h and 10-day CLP groups showed increased [^11^C]PBR28 uptake, glial cells count, and cytokine levels in the brain. Results show that sepsis modulates the gut villus length and crypt depth, alpha and beta microbial diversities, and fecal short-chain fatty acids (SCFAs). In addition, sepsis surviving animals showed a significant cognitive decline compared with the control group.

**Conclusions:**

Since several pharmacological studies have failed to prevent cognitive impairment in sepsis survivors, a better understanding of the function of glial cells and gut microbiota can provide new avenues for treating sepsis patients.

**Supplementary Information:**

The online version contains supplementary material available at 10.1186/s12974-022-02472-4.

## Introduction

More than 30 million people worldwide contract sepsis, a life-threatening disease that causes acute organ failure. Every year, 1.5 million patients in the United States develop sepsis, with at least 250,000 deaths [1–3]. Sepsis is caused by a bacterial, viral, fungus, or parasitic infection [3]. Consequently, survival patients can experience long-term memory impairment, decreased concentration, verbal fluency, and executive function impairments after being discharged from the hospital [2]. A longitudinal study of 1194 patients and 1520 hospitalizations evaluated long-term memory following sepsis. There was a 10.6% increase in the incidence of moderate-to-extreme neurological dysfunction in sepsis survivors. Sepsis was also associated with a threefold increase in moderate-to-extreme cognitive dysfunction [4]. Another retrospective cohort analysis of Medicare data found that 74.8% of people aged 65 and over who survived sepsis for 3 years or more have a residual functional disability, with 16.7% having a moderate-to-severe cognitive impairment [5]. These findings indicate that sepsis can function as a significant inflammatory “hit” on the brain, potentially increasing the brain's vulnerability to the development of dementia later in life [6, 7]. Sepsis triggers the host immune response causing vascular endothelial damage; consequently, the blood–brain barrier (BBB) breaks down, allowing and facilitating the entry of peripheral immune cells into the brain, which triggers or exacerbates glial cell activation and neuroinflammation [8]. Although several mechanisms are related to the host immune response to eliminate the infection, these mechanisms may also be related to sepsis-associated encephalopathy (SAE). It is unclear whether the systemic infection affects the intestinal microbiota or whether SAE is caused exclusively by a dysregulated host immune response.

We hypothesized that sepsis triggers gut bacterial translocation into the blood compartment, triggering a systemic inflammatory response that affects the brain.. To test our hypothesis, Wistar rats were subjected to in vivo [^11^C]PBR28 positron emission tomography (PET)/computed tomography (CT) imaging at 24 h and 10 days after cecal ligation and perforation (CLP), a gold standard model to study sepsis which closely is similar to the development and characteristics of human sepsis [9], or non-CLP surgeries. The radiotracer [^11^C]PBR28 utilized in this experiment binds to the translocator protein 18 kDa (TSPO), which is upregulated in activated microglia and astrocyte cells [10, 11], and neurons in the human olfactory bulb [12]. TSPO is a radioligand extensively used for inflammatory brain imaging, and it is considered a marker of neuroinflammation [10, 11, 13]. The novel object recognition (NOR) and Morris water maze (MWM) tasks were performed to evaluate long-term memory and spatial memory, respectively, at 10 days after sepsis induction. In addition, we performed 16S rRNA and evaluated short-chain fatty acids (SCFAs) levels in feces. The glial cell markers in the prefrontal cortex (PFC) and hippocampus were chosen, because research has found that the hippocampus is the most vulnerable region during experimental sepsis [14], and hippocampal atrophy is also observed in sepsis survivors [15]. Insult of the PFC triggers impairments of memory, attention, and executive functions found in sepsis survivors [2, 16]. Furthermore, the ischemic lesions, a consequence of sepsis pathophysiology, are frequently found in the hippocampus and PFC [16]. The brains of the animals were evaluated with in vivo and ex vivo inflammatory markers. The gut microbiome was assessed by 16S rRNA sequencing and SCFAs levels to test our hypothesis.

## Materials and methods

### CLP and non-CLP surgeries

Male Wistar rats (50 days and 250 g) from the Charles River breeding laboratory were housed in our facility for 10 days before the experiment to allow for acclimatization. The animals were housed in a 22 °C room with a relative humidity of 45–55%, a 12 h day/light period, and food and water ad libitum. The animals were either exposed to CLP surgery, the gold standard model for studying sepsis, or sham (non-CLP) surgery as a control group [17, 18]. The rats were anesthetized with 3% isoflurane before undergoing a 3-cm midline laparotomy to expose the cecum and adjoining intestine. The cecum was tightly ligated with a 3.0 silk suture 50% below the ileocecal valve and perforated once with an 18-gauge needle to induce mid-grade sepsis. A small volume of feces was extruded through the puncture site after the cecum was squeezed. The cecum was then returned to the peritoneal cavity, and the laparotomy was closed with 3.0 silk sutures. In the sham surgery group, the rats were submitted to all surgical procedures without the ligation and puncture of the cecum. Both groups were given the necessary support (50 ml/kg NaCl 0.9%, s.c.) and antibiotics (30 mg/kg ceftriaxone and clindamycin, s.c.) shortly after CLP and non-CLP procedures, as well as 12 h afterward. The Institutional Animal Welfare Committee (AWC) of the Center for Laboratory Animal Medicine and Care (CLAMC) of the University of Texas Health Science Center at Houston (UTHealth), TX, USA, approved this research protocol (IACUC Number: AWC-17-0090).

### Study design

**Fig. 1:**
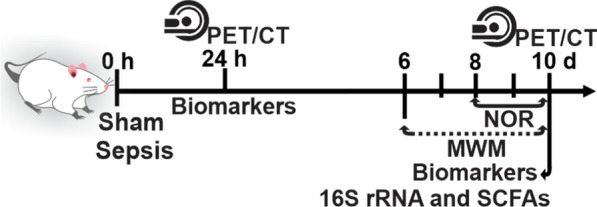
Schematic representation of timeline and experimental design. *NOR* novel object recognition, *MWM* morris water maze, 16S rRNA sequencing, and *SCFAs* short-chain fatty acids

The PET/CT scan was performed on Wistar rats at 24 h and 10 days after surgery. After the procedure, the animals were euthanized, and the brain was removed for histological and biochemical analysis. The animals in the 10-day group were subjected to behavioral tasks and then subjected to PET/CT scan procedure. After the brain imaging, the animals were euthanized, the brain was removed, and the PFC and hippocampus were dissected, flash-frozen, then preserved at − 80 °C, for subsequent biochemical and histological analysis shown in Fig. 1. We have included Additional file 1: Fig. S1,which details the number of animals used for each analysis. The survival data of rats are given in Additional file 1: Fig. S2.

### Small animal PET/CT scan

The sepsis survivors’ rats from 24-h to 10-day groups were subjected to PET/CT imaging. All animals were handled following the University of Texas MD Anderson IACUC department guidelines. The imaging was performed under 2% of isoflurane anesthesia, and PET/CT imaging was performed using a small animal Bruker Albira PET/SPECT/CT scanner (Bruker Biospin Corp., Billerica, MA, USA) with a 10 cm and a 12 cm axial field-of-view (FOV) and transaxial FOV, respectively. Cyclotron from the same facility provided the radiotracer. The radiotracer [^11^C]PBR28 was administered to rats (in 200 µL saline) via the tail vein along with the acquisition of PET/CT imaging in the list-mode. The first 20 min of recording for PET imaging was acquired, followed by a CT scan (400 µA, 45 kV, 120 projections) of the head and upper torso for attenuation correction and image registration. The list-mode data were binned into dynamic frames: 15 × 20 s, 5 × 60 s, and 2 × 300 s. The PET images were then reconstructed using the maximum likelihood expectation maximization (MLEM) method with 12 iterations. Scatter, random, decay, and attenuation corrections were applied. The analysis was performed with Pmod software (PMOD Technologies Ltd., Zürich, Switzerland). The mean standard uptake volume (SUV)-body weight (g/ml) of the whole brain was measured [19]. The SUV was calculated using the following formula: SUV = r/(a′/w), where r is the radioactivity concentration expressed as kBq/ml, which is measured by the PET scanner within a region of interest (ROI); a′ is the injected radiolabeled tracer (decay-corrected) expressed as kBq, and w is the weight the animals.

### Protein estimation

According to the manufacturer’s instructions, the protein estimation was performed using a Micro BCA^®^ protein assay kit for the sample submitted for immunoblotting, cytokine assay, and ELISA (Thermo Fischer Scientific, MA, USA) [20].

### Quantitative immunoblotting

The PFC and hippocampus of the rat brains were homogenized using Halt Protease Complete Protease Inhibitor Cocktail (100X) (Thermo Fisher Scientific, MA, USA). The homogenate was centrifuged at 12,000 rpm for 20 min at 4 °C. Protein concentrations in plasma were determined as mentioned above. Briefly, proteins were denatured in sodium dodecyl sulfate (SDS) loading buffer to a final concentration. Equal amounts of loaded proteins (30–50 μg/sample) were separated by 4–20% Mini-Protean TGX precast gels (Bio-Rad, California, USA). Then they were electrophoretically transferred to polyvinylidene fluoride membranes using a Trans-Blot^®^ Turbo™ system (Bio-Rad, California, USA) [21]. Membranes were blocked in tris-buffered saline (TBS)/ 0.05% Tween-20/ 5% milk (1 h) at room temperature (RT). Ionized calcium-binding adaptor molecule 1 (IBA-1) (unconjugated, rabbit polyclonal; 1:1000; Wako, 019-19741) and glial fibrillary acidic protein (GFAP) (1:1000, Abcam, ab7260), were added to blocking buffer, and membranes were incubated overnight at 4 °C. The next day, the membrane was washed in TBS and incubated at RT (1 h) with horseradish peroxidase-conjugated goat anti-mouse IgG secondary antibody (1:10,000) or goat–anti-rabbit IgG secondary antibody (1:10,000). After washing with TBST, bands were detected using enhanced chemiluminescence (Clarity Western ECL Substrate; Bio-Rad, California, USA) with the ChemiDoc MP (Bio-Rad, California, USA) western blotting imaging system. After imaging, the blots were incubated in the stripping buffer (Thermo Fisher Scientific; 46430, IL, USA) for 10–15 min at RT. The stripped blots were incubated in a blocking solution (5% nonfat dry milk in TBST) for 1 h and then incubated with the primary antibody directed against β-tubulin (1:5000, Abcam, ab6046) as a loading control. Densitometric analysis of each protein was conducted using Image Lab™ software (Bio-Rad, California, USA). The results were expressed as the ratio between the loading control and the target protein.

### Multiplex assay for the quantification of the inflammatory cytokines

We used multiplex fluorescent immunoassay kits (Bio-Plex Pro™ Rat Cytokine 14-Plex Assay) [22]*.* We used sensitive, specific, and widely used reagents and data collected using xMAP multiplex beads. Tissue lysates were prepared according to the instructions provided by the Bio-Plex Cell lysis kit (#171304011) with a protease inhibitor cocktail (Sigma-Aldrich, St. Louis, MO, USA), followed by centrifugation at 4 °C for 10 min at 10,000×*g*. The assays were conducted in 96-well polystyrene, round-bottom microplates. Initially, a 50 μL aliquot of the working bead mixture was transferred into the wells, and the plate was washed two times by adding 100 μL of assay buffer into each well. The standard, control, and samples at the volume of 50 μL were added to each well. The plate was incubated on a plate shaker (850 rpm) in the dark at RT for 60 min. The plate was then placed in the magnetic separator and incubated for separation for 60 s. The supernatant was carefully removed by manual inversion. The nonspecific bound antibodies were removed, and beads were washed three times by adding 100 μL of assay buffer into each well. Then, 25 μL of a detection antibody was added to each well. The plate was incubated in the dark at RT on a plate shaker (850 rpm) for 30 min, and washing was performed. The 50 μL of streptavidin-PE was added to each well as a final step. The plate was incubated on a plate shaker (850 rpm) in the dark at RT for 10 min. After removing the supernatant and performing washing, assay buffer (125 μL) was added to each well. The plate was placed onto a plate shaker for approximately 30 s to achieve gentle agitation of the beads. Samples were run in duplicates using a Bio-Plex system (Bio-Plex 200 Systems, BioRad, Hercules, CA), and data analyses were conducted in Bio-Plex Manager 4.0 using a 5-parameter logistic regression model. Cytokines interleukin (IL)-1α, IL-1β, IL-4, IL-6, IL-7, IL-10, IL-12, IL-13, IL-17, IL-18, tumor necrosis factor (TNF)-α, and interferon (IFN)-γ were evaluated in the PFC and hippocampus at 24 h and 10 days after CLP, and non-CLP surgeries.

### ELISA

We used ELISA kits for the assays of cardiolipin (MBS721201), caspase-3 (MBS018987), and caspase-9 (MBS088765). All kits were obtained from MyBioSource. The protocol was followed according to the manufacturer’s instructions.

### Immunofluorescence

For the immunofluorescence (IF) assay, the brain was fixed in 4% buffered formaldehyde solution and embedded in optimum cutting temperature (OCT). The coronal sections were cut to 8 µm using Cryostat NX70 (Thermo Scientific). Sections were blocked in 3% bovine serum albumin (BSA), horse serum, and 0.01% Triton X. After blocking, the sections were incubated overnight with antibodies for IBA-1 (unconjugated, rabbit polyclonal) at the dilution of 1:1000 (Wako, 019-19741) and GFAP (unconjugated, rabbit polyclonal) at the dilution of 1:1000 (Abcam, ab7260). The next day, after washing, the secondary antibody Alexa Fluor Plus 488 (1:1000, Invitrogen, A13201) was added following DAPI application to each section to stain nuclei. We used the following controls, primary antibody controls, secondary antibody controls, and label (endogenous tissue background) controls. The tissue sections were imaged using the confocal laser scanning microscopy platform Leica TCS SP8. We used the ImageJ software to count the number of cells from confocal images (https://imagej.nih.gov/ij/).

### Gut histopathology

Samples for histopathology were removed from the small intestine (ileum) region and placed into neutral buffered formalin for fixation, and 5 µm thick hematoxylin–eosin stained sections were prepared following paraffin embedding and histological processing. The histological sections were evaluated using standard light microscopy. Intestinal villus lengths and crypt depth measurements were performed using NIS Br software and were made from photomicrographs taken at 100 × magnification in Nikon Ti–U eclipse inverted microscope (Nikon, Tokyo, Japan).

### Fecal collection and 16S rRNA sequencing

Fecal pellets were collected from the control and sepsis groups 10 days after sham or CLP surgery. The fecal samples were immediately frozen at − 80 °C and subjected to 16S rRNA sequencing. In the first step, DNA was extracted using MO BIO PowerMag Soil DNA Isolation Kit (MO BIO Laboratories), as instructed in the manufacturer protocol. Gene sequence libraries were generated using the V4 region primers 515 F and 806 R on the Illumina MiSeq platform. The 16S rRNA gene sequencing data were obtained and analyzed by QIIME2 framework core function [23]. Reads were de-noised and merged into amplicon sequence variants (ASVs) by DADA2 pipeline in R. Taxonomic annotations were also generated against DADA2-formatted training FASTA files derived from SILVA138 Database [24, 25]. Alpha diversity (within community diversity) was estimated using R package Vegan for Shannon Index [26]. Beta diversity (between communities’ diversity) matrices were used (unweighted and weighted Unifrac) to calculate dissimilarity distances among samples. Principal coordinates analysis (PCoA) distance calculation and ordination were performed in phyloseq. The Student's *t* test was used to compare alpha diversity between the groups. ALDEx2 package was also used to calculate the differential abundance of centered log-ratio transformed taxa counts, represented as effect size.

### Short-chain fatty acids quantification

Feces sample from cecum was collected from both control and sepsis groups 10 days after sham or CLP surgery. The SCFA extraction procedure was performed as described in Zhao et al. [27]. Fecal samples were resuspended in MilliQ-grade H_2_O and homogenized using MP Bio FastPrep, for 1 min at 4.0 m/s. About 5 M HCl was added to acidify fecal suspensions to a final pH of 2.0. Acidified fecal suspensions were incubated and centrifuged at 10,000 rpm to separate the supernatant. Fecal supernatants were spiked with 2-ethylbutyric acid for a final concentration of 1 mM. Extracted SCFA supernatants were stored in 2-ml gas chromatography (GC) vials with glass inserts. SCFAs were detected using GC (Thermo Trace, 1310), coupled to a flame ionization detector.

### Behavioral assessments

#### Novel object recognition test (NOR)

The NOR task tests for recognition of familiar and novel objects and involves recollection and familiarity. The successful retrieval of the contextual details accompanying the learning episode denotes recollection. Familiarity consists of identifying the item that was presented earlier [28]. The open-field chamber was used for the NOR test. On day 8, after CLP or non-CLP, all rats were submitted to a habituation session. Animals were placed in the open field (60 × 40 cm) surrounded by 50 cm high plexiglass walls and freely explored for 5 min. On day 9, the acquisition trial was performed by exposing the rats to two identical objects for 10 min. The objects used in this experiment were square wooden cases of the same color. The heights of the objects were comparable, and the objects were heavy enough to ensure that the animals would not displace them. On day 10, in the retention trial, one of the square wooden cases was replaced by a novel object, and the rat was allowed to explore the familiar and novel object in the test box for 5 min [29]. The recognition index was calculated for each animal and was reported as the ratio TB/(TA + TB) (TA = time spent exploring the familiar object, A; TB = time spent exploring the novel object, B).

#### Morris water maze (MWM)

Testing for spatial memory test was performed using the MWM [30]. The MWM is performed in a circular pool with a diameter of 170 cm and a wall height of 65 cm. Briefly, the pool was filled with water to approximately 35 cm high, a platform (12.5 cm diameter and 33 cm height) was kept in the pool, and the water temperature was maintained at 20 ± 2 °C to avoid hypothermia. The pool was divided into four equal quadrants, and a platform was kept in the fourth, also called a target quadrant. Rats from both groups were given four trials each day during daily acquisition sessions across 4 days consecutively. A trial started as soon as the rats were placed in the tank, facing the walls. Each of the four starting points was used once in trials. The trial ends as soon as the rat reaches the platform or when 120 s elapse. If rats could not find the platform within 120 s, they were guided to the platform gently and allowed to stay there for 10 s. Soon after the session, the animals were gently dried with a clean towel and returned to the home cage. The time rats took to reach the platform was noted in all the trials. The hidden platform was removed from the tank during the probe or retention trial, and the rats were given 120 s to find the platform. The time spent swimming in the pool quadrant, where the platform had been placed was recorded.

### Statistical analysis

All the results were expressed as the mean ± standard error of the mean (SEM). Unpaired Student’s *t* tests analyzed the differences between groups. One-way ANOVA was used to get the statistical difference in exploration time in NOR. Repeated-measures ANOVA was used to determine the significance of differences in latency to find the hidden platform in MWM training. The statistically significant results are indicated by *p < 0.05. All statistical analyses were performed using GraphPad Prism 8.0 (GraphPad Software, Inc., La Jolla, California, USA). The 16S rRNA sequencing was analyzed using Kruskal–Wallis test with post hoc Benjamini–Hochberg correction.

## Results

### Sepsis increased glial cell activation in vivo, as shown by increased [^11^C]PBR28 uptake

**Fig. 2:**
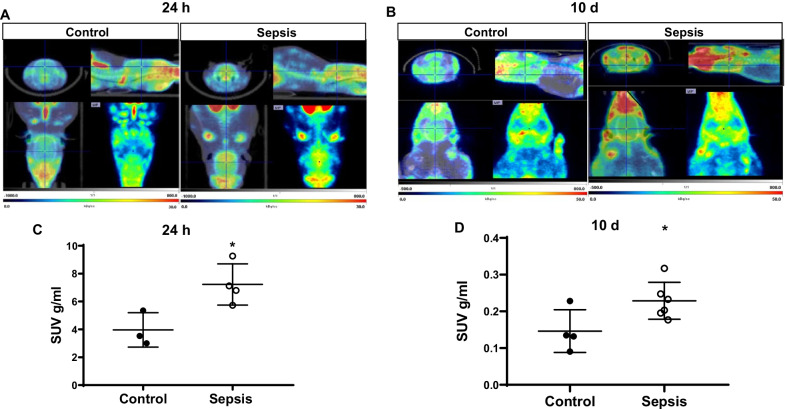
In vivo PET imaging using TSPO specific radiotracer [^11^C]PBR28. Representative summed images from the dynamic reconstruction of simultaneously acquired PET/CT scans from Wistar rats subjected to experimental sepsis by cecal ligation and perforation (CLP) and controls, 60 min after intravenous injection [^11^C]PBR28. In the experimental sepsis model, the brain’s coronal, sagittal, and dorsal orientations show increased brain [^11^C]PBR28 uptake than in controls. **A** Twenty-four hours and **B** Ten days after experimental sepsis induction. The SUV scale represents the standardized uptake value (SUV). Quantification of [^11^C]PBR28 uptake detected in the experimental sepsis model was significantly higher compared to control based on measures of brain/muscle. **C** SUV 24 h after experimental sepsis induction. **D** SUV 10 days after experimental sepsis induction. The results are expressed as the mean ± SEM for *n* = 3–6 rats. *p < 0.05 compared to controls

The sepsis survivors and sham groups from 24-h to 10-day groups were subjected to the non-invasive TSPO–PET scan followed by CT imaging. The sepsis survivors’ brain uptake of [^11^C]PBR28 was increased both times at 24 h (Fig. 2A) and 10 days (Fig. 2B) after CLP surgery compared with the sham non-CLP group. Also, Fig. 2C, D demonstrated the SUV uptake at 24 h and 10 days (*p* < 0.05), respectively.

### Sepsis increased the levels of cytokines

**Fig. 3:**
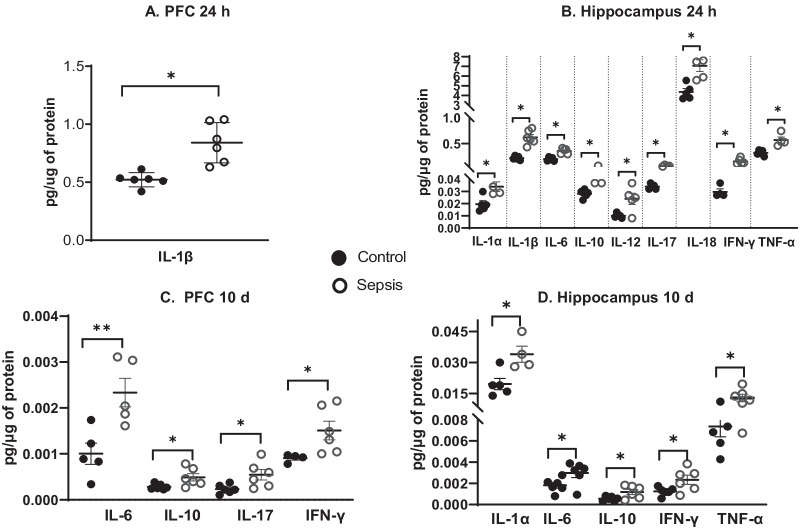
BioPlex determined the cytokine levels in the PFC and hippocampus. We evaluated the levels of cytokines (IL1-α, IL1-β, IL-4, IL-6, IL-7, IL-10, IL-12, IL-13, IL-17, IL-18, TNF-α, and INF-γ) in the **A** PFC and **B** Hippocampus at 24 h after CLP and non-CLP surgeries. **C** PFC and **D** Hippocampus at 10 days after CLP and non-CLP surgeries. The results are expressed as the mean ± SEM for *n* = 4–6 rats. *p < 0.05 and **p < 0.01 compared to controls

At 24 h after sepsis induction we found increased levels of IL-1β (*p* < 0.05) in the PFC (Fig. 3A), and higher levels of IL-1α (*p* < 0.05), IL-1β (*p* < 0.05), IL-6 (*p* < 0.05), IL-10 (*p* < 0.05), IL-12 (*p* < 0.05), IL-17 (*p* < 0.05), IL-18 (*p* < 0.05), IFN-γ (*p* < 0.05), and TNF-α (*p* < 0.05) in the hippocampus (Fig. 3B) in sepsis group compared with the sham non-CLP surgery group. Also, we evaluated the levels of cytokines in the PFC and hippocampus at 10 days after CLP and non-CLP surgeries. We found increased levels of IL-6 (*p* < 0.01), IL-10 (*p* < 0.05), IL-17 (*p* < 0.05), and IFN-γ (*p* < 0.05) in the PFC (Fig. 3C), and higher levels of IL-1α (*p* < 0.05), IL-6 (*p* < 0.05), IL-10 (*p* < 0.05), IFN-γ (*p* < 0.05), and TNF-α (*p* < 0.05) in the hippocampus (Fig. 3D), compared with the sham non-CLP surgery group. The cytokine those were not significantly different between the groups were given as Additional file 1: Figs. S3 and S4.

### Sepsis increased the GFAP, IBA-1, and TSPO protein levels in the PFC and hippocampus

**Fig. 4:**
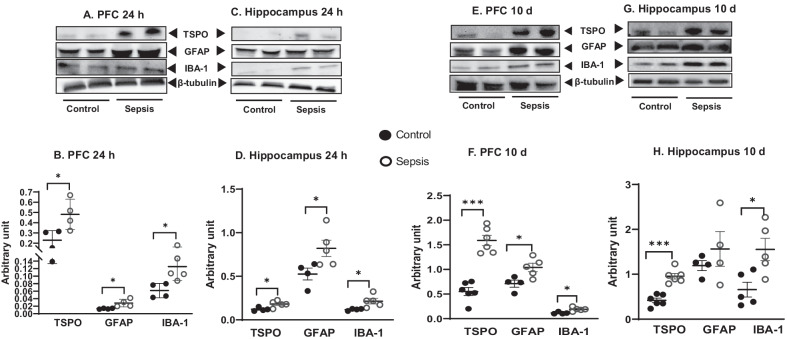
Upregulation of glial marker at 24 h **A** PFC and **C** Hippocampus; and 10 days **E** PFC and **G** Hippocampus after experimental sepsis induction in the Western blot analysis. Quantification of immunoblot data using the densitometric analysis of each protein was carried out using Image Lab™ software (Bio-Rad, California, USA). Representative immunoblots and quantification for 24 h, IBA-1, GFAP, and TSPO in the PFC **B** and hippocampus **D**. Representative immunoblots and quantification for 10 days, IBA-1, GFAP, and TSPO in the PFC **F**, and hippocampus **H**. The values for all protein levels were normalized to those of β-tubulin. The results are expressed as the mean ± SEM for *n* = 4–6 rats. *p < 0.05 and ***p < 0.001 compared to controls

**Fig. 5:**
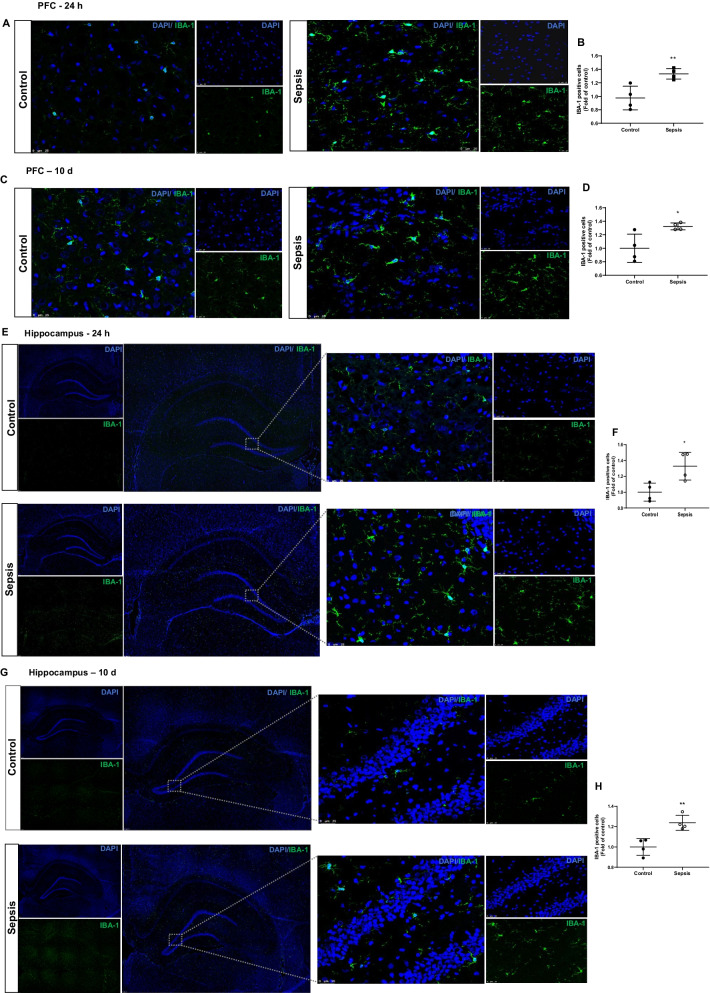
Increased microglial positive cells at 24 h and 10 days after experimental sepsis induction in PFC and hippocampus. Representative microscopic field images (magnification, × 400) immunostained with IBA-1 antibodies in the PFC **A** 24 h, **B** quantification of 24 h, **p < 0.01, **C** 10 days, **D** quantification of 10 days, *p < 0.05, after experimental sepsis induction. Representative microscopic field images (magnification, × 100 and × 400) immunostained with IBA-1 antibodies in the hippocampus **E** 24 h, **F** Quantification of 24 h, *p < 0.05, **G** 10 days, **H** Quantification of 10 days, **p < 0.01, after experimental sepsis induction. The results are expressed as the mean ± SEM for *n* = 4 rats

**Fig. 6:**
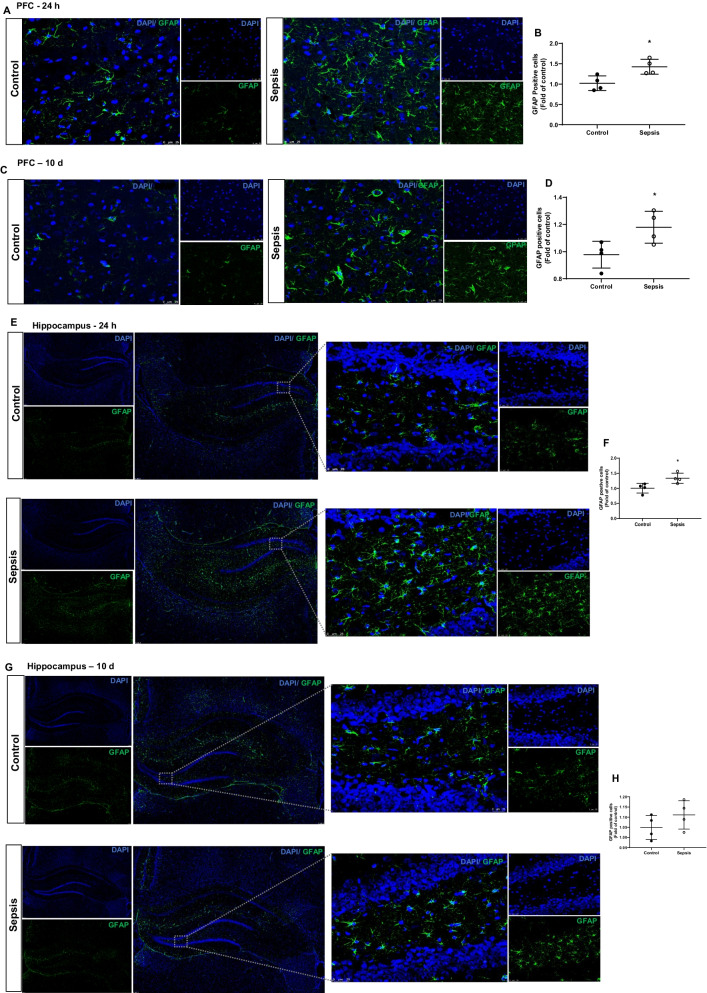
Astroglial cells at 24 h and 10 days after experimental sepsis induction in PFC and hippocampus. Representative microscopic field images (magnification, × 400) immunostained with GFAP antibodies in the PFC **A** 24 h, **B** Quantification of 24 h, *p < 0.05, **C** 10 days, **D** quantification of 10 days, *p < 0.05, after experimental sepsis induction. Representative microscopic field images (magnification, × 100 and × 400) immunostained with GFAP antibodies in the hippocampus **E** 24 h, **F** Quantification of 24 h, *p < 0.05, **G** 10 days, **H** Quantification of 10 days, *p* ≥ 0.05, after experimental sepsis induction. The results are expressed as the mean ± SEM for *n* = 4 rats

Immunoblot data show increases in IBA-1 expression (PFC and hippocampus, *p* < 0.05), GFAP (PFC, *p* < 0.05 and hippocampus, *p* < 0.05), and TSPO (PFC and hippocampus, *p* < 0.05) at 24 h after CLP and non-CLP surgery (Fig. 4A–D). At 10 days after sepsis, the data shows significant increases in the IBA-1 (PFC and hippocampus, *p* < 0.05), GFAP (PFC, *p* < 0.05 and hippocampus, *p* ≥ 0.05), and TSPO (PFC and hippocampus, *p* < 0.001). The changes in IBA-1 and GFAP in the PFC and hippocampus following sepsis were further confirmed by IF analysis. IBA-1 positive cells increased at 24 h (Fig. 5A, B; *p* < 0.01) and 10 days (Fig. 5C, D; *p* < 0.05) in the PFC after sepsis. Also, we found significant increases in IBA-1 positive cells in the hippocampus at 24 h (Fig. 5E, F; *p* < 0.05) and 10 days (Fig. 5G, H; *p* < 0.01) after sepsis. In the sepsis group, we observed increased GFAP positive cells at 24 h (Fig. 6A, B; *p* < 0.05) and 10 days (Fig. 6C, D*p* < 0.05) in the PFC. Similarly, GFAP positive cells increased at 24 h after sepsis in the hippocampus (Fig. 6E, F; *p* < 0.05) but not after 10 days (Fig. 6G, H*p* ≥ 0.05).

### Sepsis increased caspase-3 and caspase-9 protein levels in the PFC and hippocampus

**Fig. 7:**
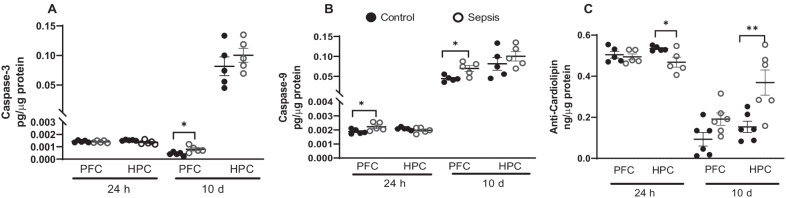
Cardiolipin and caspase levels at 24 h and 10 days after experimental sepsis were measured using ELISA in PFC and hippocampus. At 24 h and 10 days, tissue protein levels of **A** caspase-3, **B** caspase-9, and C. cardiolipin at PFC and hippocampus in experimental sepsis and sham control rats. The results are expressed as the mean ± SEM for *n* = 5–6 rats. *p < 0.05 and **p < 0.01 compared to controls

We examined the protein levels of caspase-3, caspase-9, and cardiolipin in the PFC and hippocampus 24 h and 10 days after sepsis. In the PFC, as shown in Fig. 7, we found significant increases in caspase-3 at 10 days after CLP (Fig. 7A, *p* < 0.05), whereas caspase-9 was significantly increased at both 24 h (Fig. 7B; *p* < 0.05) and 10 days (Fig. 7B; *p* < 0.05) after CLP at PFC. No change in the levels of caspase-3 and caspase-9 were found in the hippocampus after sepsis. Cardiolipin protein levels were found to decrease at 24 h (Fig. 7C; *p* < 0.05), but increased at 10 days (Fig. 7C; *p* < 0.01) in the hippocampus after CLP.

### Altered microbiome profile after sepsis

**Fig. 8:**
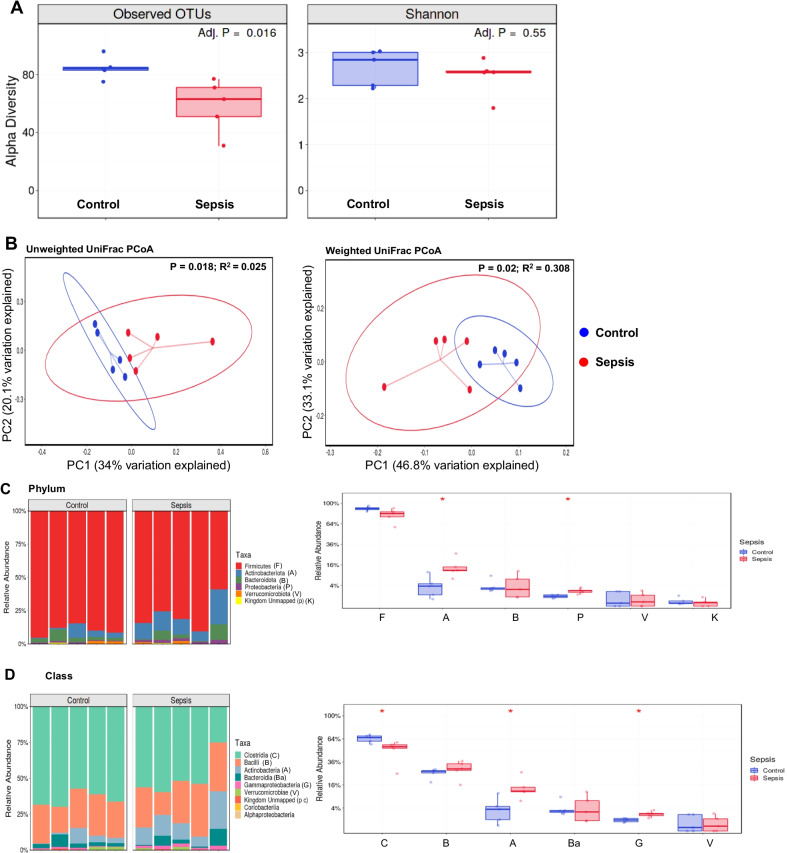
Results from 16S rRNA sequencing. **A** Alpha diversity, Observed OTUs adjusted *p* = 0.016 and Shannon index adjusted *p* = 0.55. **B** Beta diversity, unweighted unifrac PCoA *p* = 0.018; weighted unifrac PCoA *p* = 0.02. **C** Relative abundance in phyla. The phylum Actinobacteria *p < 0.05, and Proteobacteria *p < 0.05 increased in the sepsis group. **D** Relative abundance in class. The class Clostridia decreased *p < 0.05, Actinobacteria *p < 0.05 and Gammaproteobacteria *p < 0.05 classes increased in the sepsis group. The results are expressed as the mean ± SEM for *n* = 5 rats

Different statistical tests evaluate microbiome composition. Operational taxonomic units (OTUs) are identified based on sequencing data and may be characterized at multiple levels of resolution (phylum, class, order, family, genus, and species) [31]. The Shannon–Weaver diversity indices commonly measure bacterial diversity based on OTUs. UniFrac, along with standard multivariate statistical techniques, including principal coordinate analysis (PCoA), identifies factors that explain differences between microbial communities [32]. In this study, we present weighted and unweighted UniFrac values. The unweighted UniFrac distance considers only species presence and absence information and counts the fraction of branch length unique to either community. Moreover, a weighted UniFrac distance uses species abundance information and weights the branch length with abundance difference [33]. OTUs evaluated alpha diversity indices and demonstrated a decrease in richness in the sepsis group compared to the sham group (Fig. 8A; *p* = 0.016); the Shannon index did not differ between the groups (Fig. 8A; *p* = 0.55). The beta diversity, the variance between groups, decreased in the sepsis group compared to the control (Fig. 8B, unweighted, *p* = 0.018, *R*-Squared: 0.205, and weighted *p* = 0.02, *R*-Squared: 0.308). The phylum Actinobacteria and Proteobacteria increased in the sepsis group (Fig. 8C, *p* < 0.05 and *p* < 0.05, subsequently). The class Clostridia decreased (Fig. 8D, *p* < 0.05), Actinobacteria and Gammaproteobacteria classes increased in the sepsis group (Fig. 8D, *p* < 0.05 and *p* < 0.05, subsequently), and the genus did not change between the groups (*p* > 0.05, data not shown).

### Changes in spleen and decreased SCFAs levels in sepsis

**Fig. 9:**
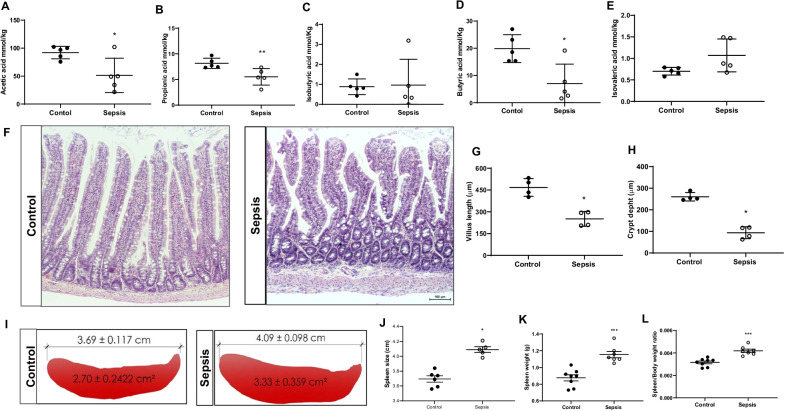
SCFAs evaluation. The levels of SCFAs were measured from cecum fecal samples **A** Acetic acid, **B** Propionic acid, **C** Isobutyric acid, **D** Butyric acid, and **E** Isovaleric acid. The levels of acetic acid, *p < 0.05; propionic acid, **p < 0.01; and butyric acid, *p < 0.05 significantly reduced after sepsis. The results are expressed as the mean ± SEM for *n* = 5 rats. **F** Gut H&E staining **G** Villus length, *p < 0.05, and **H** Crypt depth, *p < 0.05 decreased at 10 days after the sepsis (scale ba*r* = 100 μm; magnification, × 100) The results are expressed as the mean ± SEM for *n* = 4 rats. **I** and **J** Spleen size, **K** Spleen weight, and **L** Spleen/body weight ratio, ***p < 0.001, significantly increased 10 days after sepsis. The results are expressed as the mean ± SEM for *n* = 7–8 rats

SCFAs concentrations of acetate (Fig. 9A, *p* < 0.05), propionate (Fig. 9B, *p* < 0.01), and butyrate (Fig. 9D, *p* < 0.05), decreased in the sepsis group compared to the control. There was no significant difference between isobutyric acid and isovaleric acid between the sepsis and control groups (Fig. 9C and E, *p* > 0.05). The ileum villus length and crypt depth were decreased in the sepsis group compared to the control group (Fig. 9F–H, *p* < 0.05). Also, the sepsis group presented blunted intestinal villus (Fig. 9F). At 10 days after sepsis, the spleen size and weight significantly increased in sepsis rats compared to the control group (Fig. 9I–L; *p* < 0.001).

### Sepsis-induced cognitive impairment

**Fig. 10:**
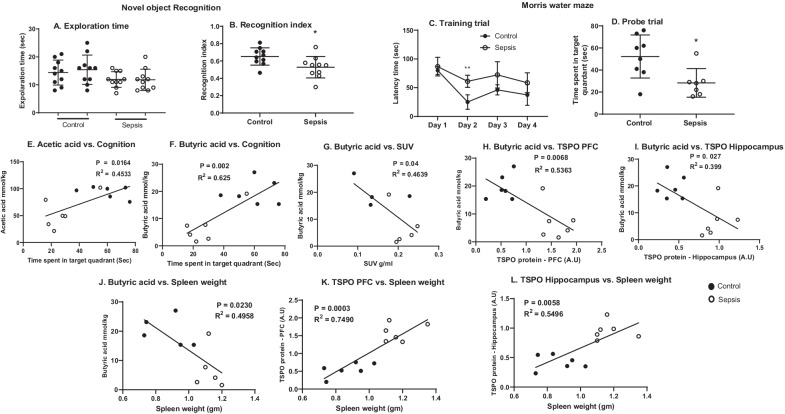
Ten days after the induction of experimental sepsis, Wistar rats were subjected to novel object recognition (NOR) test. **A** Locomotor activity and B. Recognition index. The recognition index decreased in the sepsis group compared to the control group, *p < 0.05. Evaluation of spatial memory task by Morris water maze. **C** Training trial, and **D** Probe trial. On day 2 of the training trial sepsis group taken more time to reach the platform as compared to control group, **p < 0.01. In the probe trial, sepsis-subjected rats demonstrated less time spent in the target quadrant, *p < 0.05, and *n* = 7–10 rats. The Pearson correlation between **E** Acetic acid vs. cognition, **F** Butyric acid vs. cognition, **G** Butyric acid vs. SUV uptake, **H** Butyric acid vs. TSPO at PFC, and **I** Butyric acid vs. TSPO in hippocampus, **J** Spleen weight vs. butyric acid, **K** Spleen weight vs. TSPO at PFC, **L** Spleen weight vs. TSPO at hippocampus, *n* = 4–6 rats. The results are expressed as the mean ± SEM

Ten days after rats were subjected to experimental sepsis, the animals spent less time with the novel object, showing a significantly decreased recognition index (Fig. 10B, *p* < 0.05) than the control group. However, there was no significant difference in exploration time between the two groups (Fig. 10A, *p* ≥ 0.05). In the MWM, on the day of two training trials, the sepsis group significantly increased (Fig. 10C, *p* < 0.01) the time taken to reach the platform compared to the control group, as shown in Fig. 10C. In the probe trial, the sepsis rat group spent less time in the target quadrant, evidencing spatial memory dysfunction (Fig. 10D, *p* = 0.05). Interestingly, at 10 days after the sepsis induction the correlation between the probe trial vs. the acetic acid and butyric acid had a positive correlation (Fig. 10E; *r* = 0.673, *p* = 0.0164, *R*^2^ = 0.4533 and Fig. 10F; *r* = 0.791, *p* = 0.002, *R*^2^ = 0.625, respectively).

The butyric acid had a negative correlation with SUV uptake (Fig. 10G; *r* = − 0.681, *p* = 0.04, *R*^2^ = 0.4639), and with TSPO expression in the PFC (Fig. 10H; *r* = − 0.6317, *p* = 0.0068, *R*^2^ = 0.5363) and hippocampus (Fig. 10I; *r* = − 0.732, *p* = 0.027, *R*^2^ = 0.399). Similarly, we also found an intriguing correlation between the changes in the spleen weight. At 10 days after sepsis, spleen weight had negative correlation with butyrate levels (Fig. 10J; *r* = − 0.7041, *p* = 0.023, *R*^2^ = 0.4958), probe trail (*r* = − 0.5281, *p* = 0.0430, *R*^2^ = 0.2789, data not shown) and positive correlation with TSPO expression in the PFC (Fig. 10K; *r* = 0.8855, *p* = 0.0003, *R*^2^ = 0.7490) and hippocampus (Fig. 10L; *r* = 0.7413, *p* = 0.0058, *R*^2^ = 0.549).

## Discussion

In the present study, we used the CLP surgery to induce experimental sepsis that originates polymicrobial infection from the abdominal cavity, accompanied by bacterial penetration into the bloodstream, generating a systemic inflammatory response [34]. The animals were treated with antibiotics and received fluid resuscitation to resemble human sepsis progression and characteristics. TSPO expression was evaluated using PET/CT imaging with a specific radioligand, [^11^C]PBR28, to identify in vivo glial cell activation. Both sepsis survivor groups from 24 h to 10 days presented an increased glial cell activation in the whole brain. Also, after collecting the rats’ brains, IBA-1 (microglia marker) and GFAP (astrocyte marker) positive cells were quantified and localized by IF. These markers were upregulated during the activation of these cells, and IBA-1 does not cross-react with neurons and astrocytes. Thus, our study demonstrates in vivo and ex vivo microglia and astrocyte cells activation in sepsis survivors. We previously reported that after recovery, a similar outcome in a preclinical model of pneumococcal meningitis using [^11^C]PBR28/PET imaging, where the rats demonstrated microglia and astrocyte cells activation as well as significant TSPO uptake [21]. Another study found reactive microgliosis post-injury in an ischemic stroke rat model, which correlated with [^11^C]PBR28/PET findings [35]. On the contrary, the immunological response of the human brain following abdominal surgery showed a decrease in [^11^C]PBR28 binding to the grey matter at 3–4 days postoperatively compared to baseline, and there was no correlation between [^11^C]PBR28 binding and plasma inflammatory mediator levels demonstrating that those peripheral cytokines cannot activate glial cells without brain involvement [36].

A prospective postmortem study showed a positive marker for macrophage, dendritic cell, and microglia in the hippocampus, putamen, and cerebellum of sepsis patients compared to the control subjects [37]. In a case series, sepsis postmortem brain tissues showed GFAP expression in all of them [38]. The SAE was examined in twelve cases of a 10-year retrospective study. Four out of twelve patients showed an increased number of astrocyte and microglial cells in the cerebral cortex [39]. In another study, microglial cells expressed a resident marker, the *tmem110* gene, in the white matter of septic patients, with minimal activation in the grey matter compared to control patients [40], demonstrating the involvement of the glial cells in the sepsis pathophysiology. The outcome of our systematic review also showed the glial cell activation in sepsis patients post mortem samples in the acute phase of the disease [41].

As expected, cytokines presented increased levels in 24 h and 10 days in the brain of sepsis survivors' animals. However, the most remarkable is the increase of IL-1β, IL-6, IFN-γ, and TNF-α expression in the hippocampus and IL-6, IL-17, and IFN-γ in the PFC 10 days after CLP surgery when the blood culture was negative, and the animals presented a full recovery from sepsis. These animals also demonstrated impairment of new object recognition memory. Studies have evaluated the retrograde effects of the hippocampus lesion on NOR, and they supported that the hippocampus participates in the encoding and consolidation of object memory [42]. Thus, these high levels of cytokines may be associated with the cognitive impairment triggered by sepsis. We demonstrated that sepsis keeps increased cytokines in the brain of survivor animals.

The neuronal apoptosis, evaluated using anti-caspase 3, and vascular inducible nitric oxide synthase (iNOS) expression was higher in the sepsis patients’ group; however, the TNF-α expression did not differ between groups [43]. Dr. Sharshar’s team evaluated the brain of 19 patients who died from septic shock, seven from non-septic shock, and five who died unexpectedly from extracranial damage. Ischemic, neuronal, and microglial apoptosis scores differed between groups and were more significant in septic shock patients than in non-septic shock patients and extracranial injury-related fatalities [44]. Our study also found an increased expression of the caspase-3, and caspase-9 in the PFC and changes in cardiolipin in hippocampus of sepsis survivors’ animals. Sepsis triggered the expression of TSPO, and the pro-apoptotic function of TSPO may involve the modulation of the channel formed by the mitochondrial voltage-dependent anion channel (VDAC) and the adenine nucleotide transporter (ANT) [45, 46]. Sepsis increased the expression of the TSPO in our research. Its high expression has also been linked to inflammation/infection and triggers cell death via caspase activation [46]. In a previous study, modulation of neuroinflammation during sepsis by blocking TSPO using an antagonist decreased brain cytokine expression and prevented cognitive impairment [45].

Furthermore, TSPO expression was also found upregulated in several inflammatory [21] and neurodegenerative diseases, such as Alzheimer’s disease [47], Parkinson’s disease [48], and pediatric autoimmune neuropsychiatric disorders associated with streptococcal infection (PANDAS) [49]. Although we found parallel changes in inflammatory markers and cognitive function, additional studies are warranted to examine whether inhibition of glial cell activation could prevent sepsis-induced cognitive impairment. The coadjuvant treatment of the TSPO antagonist, PK-11195, improved mitochondrial function and reduced microglial activation and cognitive impairment [45]. Changes in spleen weight often refer the alterations in the immune system. Earlier studies have reported CLP-induced splenomegaly in murine sepsis survivors [50]. Corroborating with the results, we found significantly increased spleen weight 10 days after CLP. We identified a significant variation in the fecal microbial diversity across the groups throughout the research. We observed at several taxonomic levels alpha diversity and relative abundances of particular gut bacterial RNA. For example, the relative abundance of Phylum Actinobacteria and Proteobacteria, classes Actinobacteria and Gammaproteobacteria, were more significant in the sepsis group than in the control group.

The specific processes that cause an increase in *Proteobacteria* during diseases, especially during inflammation, are unclear [51]. The phylum Proteobacteria’s genus *Bilophila* was more prevalent in fecal samples of Alzheimer’s disease patients than in healthy controls [52]. Compared to healthy control, patients with Parkinson’s disease showed increased *Proteobacteria* and *Enterobacteriaceae* in the feces [53]. Also, the abundance of *Proteobacteria* was highly increased in post-stroke cognitive impairment patients compared with post-stroke non-cognitive impairment patients [54]. A preclinical model of spinal cord injury [55] and ischemic stroke [56] led to an increased abundance of *Proteobacteria*, demonstrating that CNS injury affects the gut microbiota. Sepsis is a systemic infection that also affects the CNS, decreasing cognition, and has a component that modulates the gut microbiome.

The class Clostridia levels, a producer of SCFAs, decreased in the sepsis group. Alzheimer’s disease patients [52], the elderly population [57], and also patients with amnestic mild cognitive impairment [58] presented a decrease in *Clostridia* levels in the fecal samples. On the other hand, *Clostridiacea* exerted a neuroprotective effect in a mouse model of traumatic brain injury [59].

The acetic, propionic, and butyric acids levels decreased in the sepsis group 10 days after sepsis induction. SCFA levels and their producers are critical for brain homeostasis. For example, colonization of germ-free mice with the butyrate producer *Clostridium* decreased BBB permeability, and butyrate was related to increased occludin expression in the frontal brain and hypothalamus [60]. Extensive defects in microglia were also observed in germ-free mice, with altered cell proportions and an immature phenotype, diminishing microglia immune response. Treatment with sodium propionate, sodium butyrate, and sodium acetate or three strains of altered Schaedler flora, including *Bacteroides distasonis*, *Lactobacillus salivarius*, and *Clostridium cluster* restored defective microglia [61]. SCFAs can also regulate microglia by activating the aryl hydrocarbon receptor (AHR), reducing glial activation as well as CNS inflammation and neurodegeneration [62]. Sodium butyrate administration reversed the cognitive impairment and the histone deacetylase (HDAC) activity in the prefrontal cortex and hippocampus 10 days after experimental sepsis [63]. Also, sodium butyrate treatment re-established brain-derived neurotrophic factor (BDNF) and glial cell line-derived neurotrophic factor (GDNF) expression, preventing memory impairment in experimental pneumococcal meningitis [63].

## Conclusions

Our study demonstrated that the acute-phase response of sepsis increased TSPO uptake and glial cell activation, and by the end of 10 days after surgery, the antibiotic treatment and the host immune response cleared the active source of infection. Nonetheless, the glial cell remained activated, the animals presented cognitive impairment, and the topography of neuroinflammation in the brains of animals could be determined. The animals also showed intestinal dysbiosis assessed through a decrease in the villus length and crypt depth, a decrease in microbiota diversity and abundance, and a reduction in SCFAs’ primary metabolites produced by the gut microbiota. Sepsis has a strong link with inflammation, and several drug trials failed to prevent long-term cognitive impairment; maybe understanding the function of the microbiota–gut–brain axis is a new avenue to develop treatment strategies for patients who recovered from sepsis.

## Supplementary Information

**Additional file 1:** Supplementary figures.

## Data Availability

The datasets during and/or analyzed during the current study are available from the corresponding author on reasonable request.

## Abbreviations

AHR: Aryl hydrocarbon receptor
ANT: Adenine nucleotide translocator
BBB: Blood–brain barrier
BSA: Bovine serum albumin
BCA: Bicinchoninic acid
BDNF: Brain-derived neurotrophic factor
CLP: Cecal ligation and puncture
CNS: Central nervous system
CT: Computed tomography
FOV: Field-of-view
GC: Gas chromatography
GFAP: Glial fibrillary acidic protein
GDNF: Glial cell line-derived neurotrophic factor
HDAC: Histone deacetylase
IL: Interleukin
IFN: Interferon
iNOS: Inducible nitric oxide synthase
IBA-1: Ionized calcium-binding adaptor molecule 1
MLEM: Maximum likelihood expectation maximization
MWM: Morris water maze
NOR: Novel object recognition
OTU: Operational taxonomic unit
PBR: Peripheral benzodiazepine receptor
PANDAS: Pediatric autoimmune neuropsychiatric disorders associated with streptococcal infection
PCoA: Principal coordinate analysis
PET: Positron emission tomography
PFC: Prefrontal cortex
RT: Room temperature
ROI: Region of interest
rRNA: Ribosomal ribonucleic acid
SAE: Sepsis-associated encephalopathy
SDS: Sodium dodecyl sulfate
SEM: Standard error of mean
SCFAs: Short-chain fatty acids
SPECT: Single photon emission computed tomography
SUV: Standard uptake volume
TA: Time spent exploring the familiar object, A
TB: Time spent exploring the novel object, B
TSPO: Translocator protein
TNF: Tumor necrosis factor
VDAC: Voltage-dependent anion channel
