# Supplementary material for: A crosstalk between gut and brain in sepsis-induced cognitive decline
Source: J Neuroinflammation. 2022 May 23;19:114. doi: 10.1186/s12974-022-02472-4 (PMC9125851; doi:10.1186/s12974-022-02472-4)
Supplement: Supplementary file 1 — Additional file 1: Supplementary figures. [file 12974_2022_2472_MOESM1_ESM.pdf]

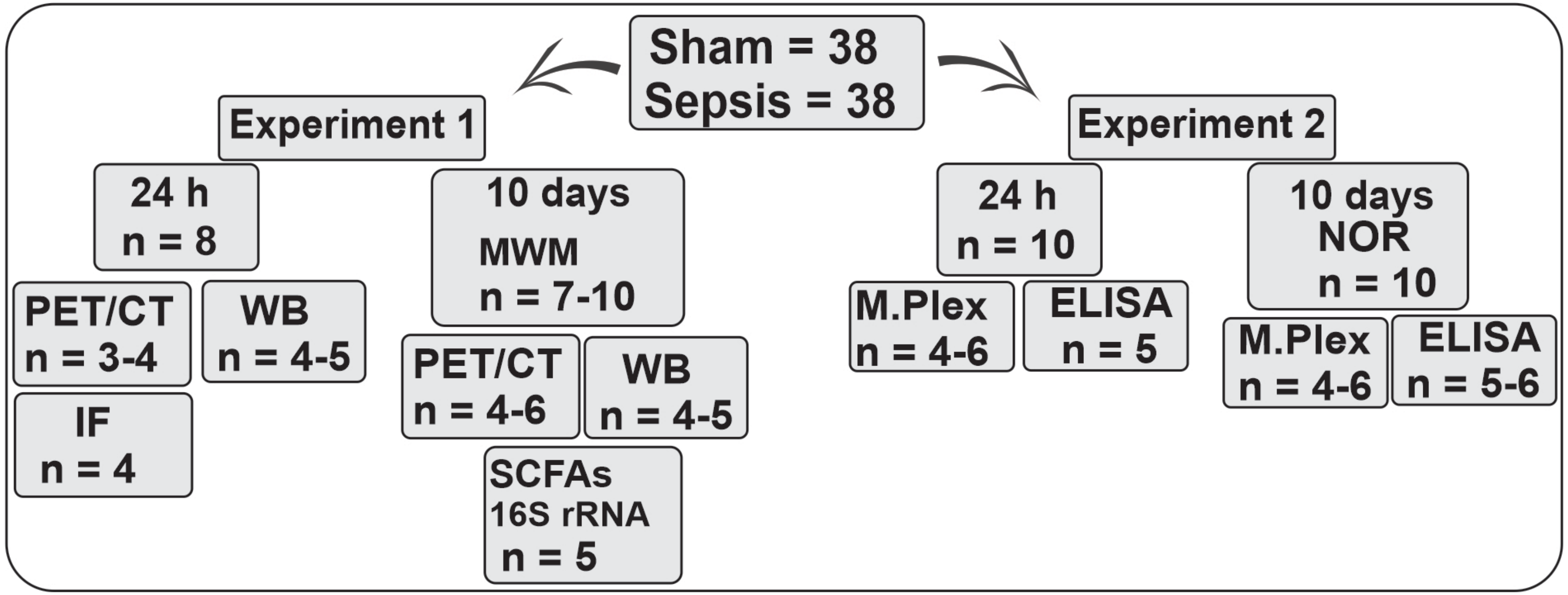

Supplementary figure 1

Flow chart of animal experiments: The number of rats used in behavioral tasks, PET/CT, and biochemical analysis.

Days	1	2	3	4	5	6	7	8	9	10
Sham n = 18	–	–	–	–	–	–	–	–	–	n = 18
Sepsis n = 30	₮₮₮	₮ ₮ ₮ ₮	₮ ₮	₮ ₮	₮	–	–	–	–	n = 18
Sham n= 20	–	–	–	–	–	–	–	–	–	n = 20
Sepsis n = 34	₮₮₮₮	₮₮₮₮₮	₮ ₮	₮ ₮	₮	–	–	–	–	n = 20

Supplementary figure 2

Survival data**:** The mortality of rats until 10 days after CLP and non-CLP (sham) surgery. ₮ represents death of one rat.

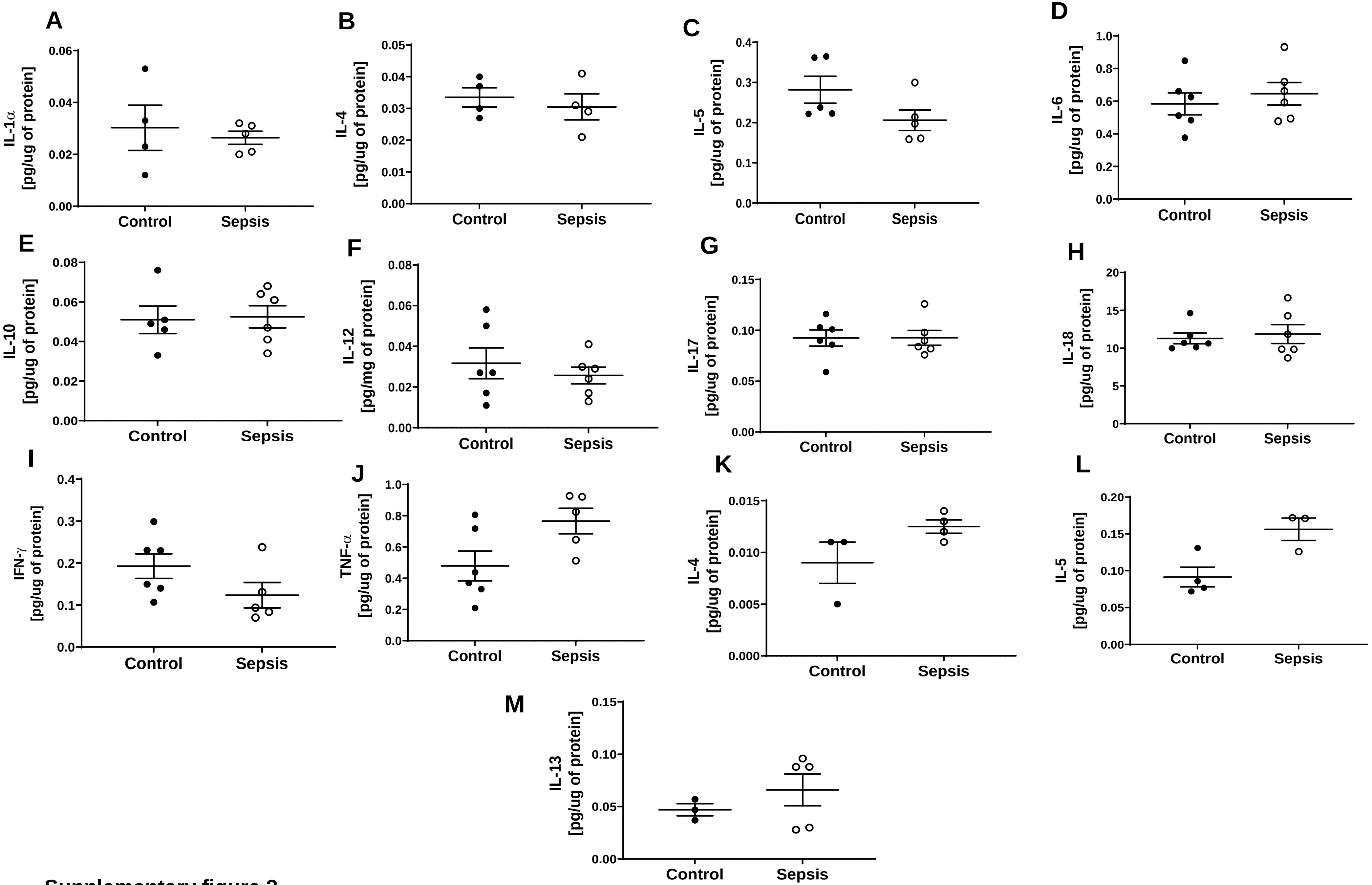

Supplementary figure 3

Cytokine levels: Cytokine levels measured using multi-plex analysis after 24 hours of CLP or non-CLP surgery at PFC (A-J) and hippocampus (K-M).

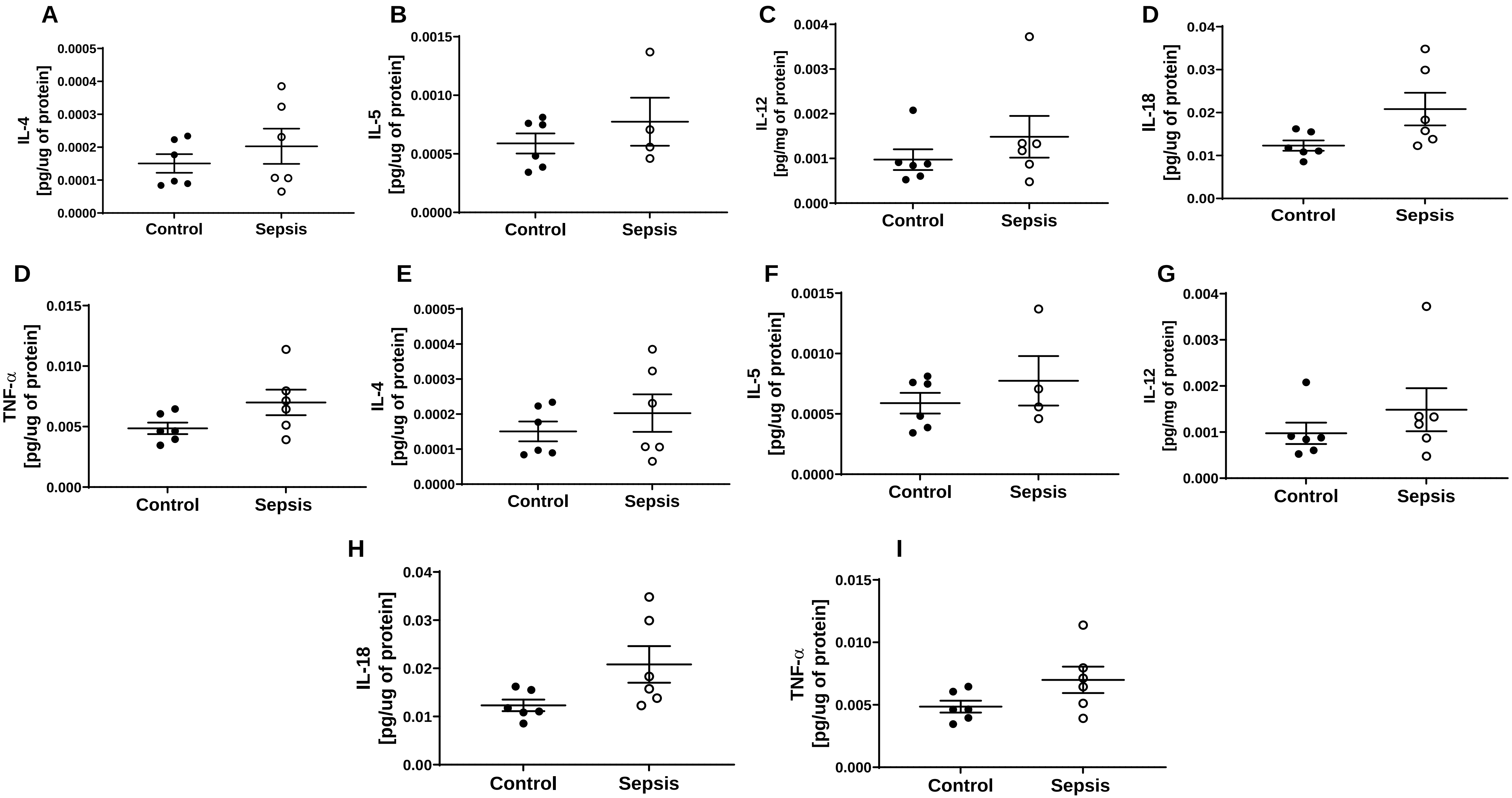

Supplementary figure 4

Cytokine levels: Cytokine levels measured using multi-plex analysis after 10 days of CLP or non-CLP surgery at PFC (A-D) and hippocampus (E-I).
